# GABA_B_ Receptors Mediate Intracellular Calcium Release in Astrocytes of the Prefrontal Cortex

**DOI:** 10.1111/ejn.70187

**Published:** 2025-07-14

**Authors:** Jennifer Bostel, Alina J. Kürten, Antonia Beiersdorfer

**Affiliations:** ^1^ Division of Neurophysiology University of Hamburg Hamburg Germany

**Keywords:** astrocytes, calcium signaling, GABA_B_ receptor, intracellular calcium stores, PLC/IP_3_‐signaling cascade, prefrontal cortex

## Abstract

The prefrontal cortex (PFC) is a cortical brain region whose multifaceted functions are based on a complex interplay between excitatory pyramidal neurons, inhibitory GABAergic interneurons, and astrocytes maintaining a fine‐tuned excitation/inhibition balance (E/I balance). The regulation of the E/I balance in cortical networks is crucial as the disruption leads to impairments in PFC‐associated behavior and pathologies. Astrocytes express specific GABA receptors that mediate intracellular Ca^2+^ signaling upon stimulation by γ‐aminobutyric acid (GABA), resulting in the release of gliotransmitters. GABA‐mediated Ca^2+^ signaling in astrocytes has been of great interest in the past; however, especially, the signaling pathway greatly varies across brain regions and from development to adulthood. Here we took advantage of GLAST‐promoter driven GCaMP6s expression in astrocytes to study GABAergic Ca^2+^ signaling, especially in young adult astrocytes of the PFC by confocal microscopy. The results show that GABA induces Ca^2+^ signaling via the stimulation of the metabotropic GABA_B_ receptor in astrocytes. GABA_B_ receptor‐mediated Ca^2+^ signals greatly depend on intracellular Ca^2+^ stores rather than on extracellular Ca^2+^. Additionally, antagonists of the PLC/IP_3_‐signaling cascade significantly reduced GABA_B_ receptor‐mediated Ca^2+^ signaling in astrocytes. Moreover, inhibition of the G_i/o_ signaling cascade did not have an effect on GABA_B_receptor‐mediated Ca^2+^ transients, suggesting that astrocytic GABA_B_ receptors in the PFC of adolescent mice are coupled to the G_q_‐GPCR signaling pathway exclusively.

## Graphical abstract

We took advantage of GLAST‐promoter driven GCaMP6s expression in astrocytes to study Ca^2+^ signaling in astrocytes of the young adult prefrontal cortex in response to GABA with confocal Ca^2+^ imaging. The results show that GABA induces Ca^2+^ signaling via the stimulation of the metabotropic GABA_B_ receptor in astrocytes. GABA_B_ receptor‐mediated Ca^2+^ signals in prefrontal cortex astrocytes greatly depend on intracellular Ca^2+^ stores and the PLC/IP3‐signaling cascade.

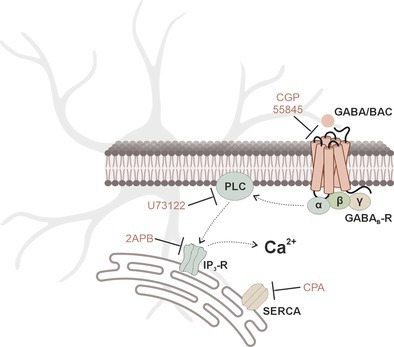

**Abbreviations**
0 Ca^2+^
: Ca^2+^‐free ACSF
2‐APB: 2‐aminoethoxydiphenylborane
ACSF: artificial cerebrospinal fluid
BAC: (R,S)‐baclofen
Ca^2+^
: calcium ion
cAMP: cyclic adenosine monophosphate
CGP: CGP 55845 hydrochloride
CNS: central nervous system
CPA: cyclopiazonic acid
D‐APV: D‐2‐amino‐5‐phosphonovaleric acid
E/I balance: excitation/inhibition balance
GABA: γ‐aminobutyric acid
GAT: GABA transporters
GECI: genetically encoded Ca^2+^ indicator
GFP: green fluorescence protein
GLAST: glutamate aspartate transporter
GPCR: G protein‐coupled receptor
IP_3_
: inositol 1,4,4‐trisphosphate
L: layer
*n*
: number of experiments
NBQX: 2,3‐dioxo‐6‐nitro‐1,2,3,4‐tetrahydrobenzo[f]quinoxaline‐7‐sulfonamide
NE: norepinephrine
NGS: normal goat serum
p: error probability
PBS: phosphate buffer saline
PFC: prefrontal cortex
PLC: phospholipase c
PTX: pertussis toxin
ROI: region of interest
TTX: tetrodotoxin
U7: U 73122
ΔF: relative changes in fluorescence

## 1 Introduction

Gamma‐aminobutyric acid (GABA) serves as the major inhibitory neurotransmitter in the central nervous system (CNS), whereas glutamate is considered to be the main excitatory neurotransmitter in vertebrates (Yoon et al. 2012). In general, there are two major classes of neurons in the prefrontal cortex (PFC), approximately 80%–90% excitatory glutamatergic pyramidal neurons and 10%–20% GABA‐releasing interneurons establishing a well‐defined neuronal circuit maintaining a fine‐tuned balance between excitation and inhibition (E/I balance) (Isaacson and Scanziani 2011; Ferguson and Gao 2018; Xu et al. 2019). The E/I balance in neuronal networks is crucial for information processing as the disruption of the E/I balance induces impairments of PFC‐associated behavior, such as working memory, social interaction, and emotional regulation (Ferguson and Gao 2018). However, not only neurons are involved in the establishment and maintenance of cellular information processing in the CNS but also astrocytes fulfill diverse functions contributing to healthy brain physiology. To do so, astrocytes contribute to and regulate the blood–brain barrier, supply neurons with metabolites, and modulate synaptic transmission in the neuronal network (Volterra and Meldolesi 2005; Allen and Barres 2009). In fact, astrocytes have been shown to sense as well as release GABA, thereby modulating synaptic transmission, making astrocytes an ideal cellular partner to contribute to the maintenance and tuning of the E/I balance in cortical networks (Liu et al. 2000; Kozlov et al. 2006; Lee et al. 2010; Pandit et al. 2020; Liu et al. 2022; Park et al. 2025). To sense GABA release by neurons, astrocytes express both types of GABA receptors, that is, the ionotropic GABA_A_ receptor as well as the metabotropic GABA_B_ receptor (Kettenmann et al. 1987; Meier et al. 2008; Mariotti et al. 2016; Mederos et al. 2021; Cheng et al. 2023; Cahill et al. 2024). Additionally, GABA transporters, such as GAT1 and GAT3, are present in astrocytes of different brain regions, regulating extracellular GABA availability (De Biasi et al. 1998; Doengi et al. 2009; Shigetomi et al. 2011; Boddum et al. 2016). GABA_A_ receptors are chloride channels mediating a chloride efflux upon GABA stimulation in astrocytes (Untiet et al. 2023). GABA_B_ receptors, on the other hand, are G_i/o_‐coupled metabotropic receptors leading to an inhibition of the adenylyl cyclase and the reduction of intracellular cyclic adenosine monophosphate (cAMP) levels. GABA‐mediated Ca^2+^ signaling in astrocytes has also been reported in multiple studies (Meier et al. 2008; Doengi et al. 2009; Mariotti et al. 2016; Cahill et al. 2024); however, the signaling pathways resulting in GABAergic Ca^2+^ responses in astrocytes show high variability across brain regions and development. In this study, we aimed to investigate the signaling pathway that induces GABAergic Ca^2+^ signaling in astrocytes of the PFC of young adult mice by confocal Ca^2+^ imaging. The results show that GABA‐evoked GABA_B_ receptor‐mediated Ca^2+^ signals in PFC astrocytes depend on intracellular Ca^2+^ stores and the PLC/IP_3_‐signaling cascade, but not the G_i/o_‐signaling cascade, indicating that astrocytic GABA_B_ receptors in the PFC are coupled to the G_q_‐GPCR signaling pathway.

## 2 Material and Methods

### 2.1 Animals and Preparation of PFC Slices

Mice of the GLAST‐Cre^ERT2^ × GCaMP6s^fl/fl^ (age: 4–6 weeks old) strain (Mori et al. 2006; Madisen et al. 2010) were kept at the institutional animal facility of the University of Hamburg. Animals were kept in a 12/12 h light cycle with food and water ad libitum. Animal rearing and all experimental procedures were performed according to the European Union's and local animal welfare guidelines (GZ G21305/591‐00.33; Behörde für Gesundheit und Verbraucherschutz, Hamburg, Germany). Both sexes were used for experiments. For induction of GCaMP6s expression controlled by the GLAST promoter, tamoxifen (Carbolution Chemicals GmbH, St. Ingbert, Germany) was dissolved in ethanol and Mygliol812 (Sigma Aldrich) and injected intraperitoneally for three consecutive days (starting p21; 100 mg/kg body weight). Animals were analyzed 14–28 days after the first injection. Mice were anesthetized using isoflurane (5% in O_2_) and decapitated. Subsequently, the PFC was removed from the opened head in cooled preparation solution (molarities in mM: 83 NaCl, 1 NaH_2_PO_4_ × 2H_2_O, 26.2 NaHCO_3_, 2.5 KCl, 70 sucrose, 20 D‐(+)‐glucose, 2.5 MgSO_4_ × 7 H_2_O). Standard artificial cerebrospinal fluid (ACSF) for experiments and storage of the preparations consisted of (molarities in mM): 120 NaCl, 2.5 KCl, 1 NaH_2_PO_4_ × 2H_2_O, 26 NaHCO_3_, 2.8 D‐(+)‐glucose, 1 MgCl, 2 CaCl_2_. The modified 0 Ca^2+^‐ACSF consisted of (molarities in mM): 120 NaCl, 2.5 KCl, 1 NaH_2_PO_4_ × 2H_2_O, 26 NaHCO_3_, 2.8 D‐(+)‐glucose, 3 MgCl, 0.5 EGTA. Preparation solution and ACSF were continuously perfused with carbogen (95% O_2_, 5% CO_2_) to maintain the pH of 7.4 and to supply oxygen.

### 2.2 Reagents

The reagents D‐2‐amino‐5‐phosphonovaleric acid (D‐APV; antagonist of NMDA receptors; #D‐145; working concentration: 100 μM), 2,3‐dioxo‐6‐nitro‐1,2,3,4‐tetrahydrobenzo[f]quinoxaline‐7‐sulfonamide (NBQX; antagonist of AMPA/kainate receptors; #N‐186; working concentration: 10 μM), tetrodotoxin (TTX; inhibiting voltage‐gated sodium channels; #T‐550; working concentration: 0.5 μM), and gabazine (antagonist of GABA_A_ and Glycine receptors, #G‐215; working concentration: 5 μM) were purchased from Alomone Labs (Jerusalem, Israel). The compounds (*R*,*S*)‐4‐amino‐3‐(4‐chlorophenyl)butanoic acid (R,S)‐baclofen; GABA_B_ receptor agonist; #ab120149; working concentration: 200 μM); and cyclopiazonic acid (CPA; Ca^2+^‐ATPase inhibitor; #ab120300; working concentration: 20 μM) were obtained from Abcam (Cambridge, UK). 2‐Aminoethoxydiphenylborane (2‐APB; IP3 receptor antagonist; #1224; working concentration: 100 μM), (2*S*)‐3‐[[(1*S*)‐1‐(3,4‐dichlorophenyl)ethyl]amino‐2‐hydroxypropyl](phenylmethyl)phosphinic acid hydrochloride (CGP 55845 hydrochloride; GABA_B_ receptor antagonist; #1248; working concentration: 10 μM), GABA (endogenous agonist of GABA receptors; #0344; working concentration: 50 μM–1 mM), and 1‐[6‐[[(17β)‐3‐methoxyestra‐1,3,5(10)‐trien‐17‐yl]amino]hexyl]‐1*H*‐pyrrole‐2,5‐dione (U 73122; phospholipase C inhibitor; #1268; working concentration: 100 μM) and (*S*)‐3,5‐dihydroxyphenylglycine (DHPG, mGluR Agonist, #0805/5, working concentration: 50 μM) were purchased from BioTechne (Wiesbaden, Germany). Pertussis toxin (PTX, G_i_‐protein receptor inhibitor; HB4729; working concentration: 7.5 μg/mL) was purchased from HelloBio (Princeton, USA). Stock solutions were prepared according to the manufacturer's instructions and dissolved in ACSF at the final concentrations immediately prior to the experiment. Agonists were applied for 30 s, whereas antagonists were applied for 10–30 min via the perfusion system.

### 2.3 Confocal Ca^2+^ Imaging, Data Analysis, and Statistics

For Ca^2+^ imaging experiments, slices of the PFC (220 μm, coronal) of GLAST‐Cre^ERT2^ × GCaMP6s^fl/fl^ mice were used. Slices were prepared using a vibratome (Leica VT1200S). Coronal PFC slices were transferred into the recording chamber, fixed with a platinum grid covered with nylon strings and continuously perfused with ACSF via the perfusion system driven by a peristaltic pump (Ismatec, Wertheim, Germany) at a flow rate of 2.35 mL min^−1^ at room temperature. Changes in cytosolic Ca^2+^ concentration in astrocytes were detected by the fluorescence of GCaMP6s (excitation: 488 nm; emission: 500–530 nm) using a confocal microscope (eC1, Nikon, Düsseldorf, Germany). Images were acquired at a time rate of one frame every 3 s. To analyze changes in cytosolic Ca^2+^ in single cells, regions of interest (ROIs) were manually defined using Nikon EZ‐C1 3.90 software including soma and processes. Astrocytes were identified by GLAST promoter‐driven GCaMP6s expression. The changes in Ca^2+^ were recorded throughout the experiments as relative changes in GCaMP6s fluorescence (ΔF) with respect to the baseline fluorescence, which was normalized to 100%. Agonists and antagonist were applied via the perfusion system using a peristaltic pump and a pump speed of 2.35 mL min^−1^. The agonist application occurred for 30 s, whereas antagonist application occurred depending on the antagonist for 10–30 min prior to the next agonist application. Experiments with the G_i_‐protein antagonist PTX were carried out by a 4 h‐preincubation with PTX diluted in ACSF in an incubation bath at room temperature. For comparison control slices were kept for 4 h in an incubation bath solely in ACSF. Quantification of the Ca^2+^ transients were calculated by the amplitude of ΔF. All values are stated as mean values ± standard error of the mean. The number of experiments is given as *n* = x/y/z, where x is the number of analyzed cells and y is the number of analyzed slices and z the number of animals. At least three animals were analyzed in all experiments. Statistical significance was estimated by comparing three means using the Wilcoxon test for paired data sets, and the Mann–Whitney *U* test for unpaired data sets. The Kruskal–Wallis ANOVA, followed by the Dunn's test was performed when comparing more than three data sets. The Grubbs test was used to identify outliers throughout all experiments. We did not test for normal distribution. The error probability *p* was *p < 0.05; **p < 0.01; ***p < 0.001.

### 2.4 Immunohistochemisty

To validate the specificity of astrocytic GCaMP6s expression in GLAST‐Cre^ERT2^ × GCaMP6s^fl/fl^ we performed double immunohistochemical stainings for GCaMP6s and the astrocyte‐specific marker S100b. Because GCaMP6s is based on the green fluorescent protein (GFP), an anti‐GFP antibody was used to detect GCaMP6s expression. Mice were prepared as described before (Section 2.1), the PFC was removed from the opened head and fixed for 2 h in 4% PFA at room temperature. PFA was removed and the PFC was washed 3 × 10 min in phosphate‐buffered saline (1 × PBS) (in mM: NaCl, 130; Na_2_HPO_4_, 7; NaH_2_PO_4_, 3). Slices from the PFC were prepared using a vibratom (VT1000, Leica, Bensheim, Germany). Slices were blocked and permeabilized with 10% normal goat serum (NGS) and 0.5% Triton X‐100 in PBS for 1 h. The following primary antibodies were used: anti‐GFP (chicken, #132006, Synaptic Systems, Göttingen, Germany) and anti‐S100b (rabbit, Z0311, Dako, Hamburg). Primary antibodies were diluted in in 1.0% NGS and 0.05% Triton X‐100 in PBS and incubated for 48 h at 4°C. The corresponding secondary antibodies (Alexa Fluor 488 goat anti‐chicken, #Ab150173, Abcam, Cambridge, UK; Alexa Fluor 555 goat anti‐rabbit, #A21429, Life Technologies GmbH, Darmstadt, Germany) were diluted in PBS and incubated for 24 h at 4°C. Slices were washed three times for 10 min in 1 × PBS. Slices were mounted with Immu‐Mount (Life Technologies GmbH) on slides and recorded using a confocal microscope (eC1, Nikon, Düsseldorf, Germany). Confocal images were adjusted to contrast and brightness using ImageJ and GIMP. For quantification of GCaMP6s‐expressing astrocytes in GLAST‐Cre^ERT2^ × GCaMP6s^fl/fl^ a cell counting tool provided by Fiji ImageJ was used. A total of 18 slices from three mice were analyzed.

## 3 Results

### 3.1 GABA Induces Ca^2+^ Signals in Astrocytes of the PFC

**FIGURE 1:**
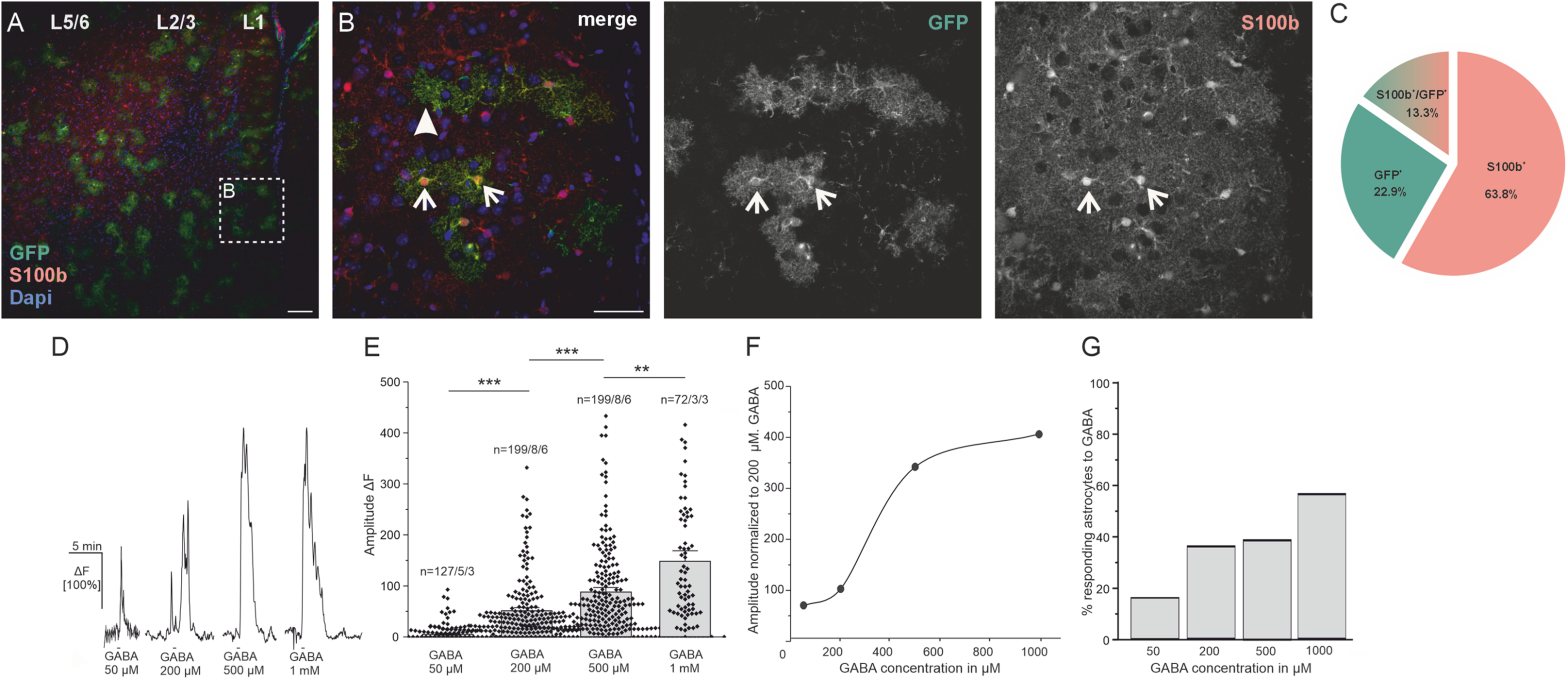
GABA induces Ca^2+^ transients in PFC astrocytes. (A) Immunohistochemical organization of the prefrontal cortex. GLAST‐Cre^ERT2^ × GCaMP6s^fl/wt^ express the genetically encoded Ca^2+^ indicator, GCaMP6s, controlled by the astrocyte‐specific GLAST promoter. In Layers 1–6 (L1–L6) of the PFC GCaMP expression (anti‐GFP, green) show co‐localization with the astrocyte marker S100b (red). Scale bar: 100 μm (B) Higher magnification image of S100b‐positive astrocytes (red) also expressing GCaMP (green) (indicated by arrows). Nuclei are stained with DAPI. Scale bar: 50 μm. (C) Quantification of GCaMP6s‐ and S100bexpressing astrocytes in GLAST‐Cre^ERT2^ × GCaMP6s^fl/fl^ mice. (D) Dose‐response relationship of Ca^2+^ transients in astrocytes to 50 μM, 200 μM, 500 μM, and 1 mM GABA. (E) Amplitudes in relative changes in fluorescence (ΔF) of Ca^2+^ transients in astrocytes to 50 μM, 200 μM, 500 μM, and 1 mM GABA. Statistical significance was determined by the Kruskal–Wallis ANOVA and the Dunns's test. The error probability *p* was ** *p* < 0.01 and ***p < 0.001. (F) Average amplitudes of GABA‐induced Ca^2+^ transients normalized to the 200‐μM GABA response. (G) Percentage of astrocytes responding to the different concentrations of GABA with Ca^2+^ transients.

To study GABAergic Ca^2+^ signaling in astrocytes of the PFC, we took advantage of GLAST‐Cre^ERT2^ × GCaMP6s^fl/fl^ mice, in which the genetically encoded Ca^2+^ indicator (GECI) GCaMP6s is expressed under control of the astrocyte‐specific GLAST promoter (Mori et al. 2006; Madisen et al. 2010). GCaMP6s is a commonly used GECI suitable for Ca^2+^ imaging in glial cells (Lohr et al. 2021). To validate astrocyte‐specific GCaMP6s expression in these mice, we performed immunohistochemical stainings using anti‐GFP antibodies to stain for GCaMP. Additionally, we used the astrocyte‐specific marker S100b (Tateishi et al. 2006). The results show co‐localization of anti‐GFP and anti‐S100b indicating astrocyte‐specific GCaMP expression in astrocytes of the PFC (Figure 1A,B see arrows). To evaluate the recombination efficiency of GLAST‐CreERT2 × GCaMP6s^fl/fl^ mice, we quantified the number of astrocytes positive for S100b and GCaMP6s in the PFC using a semi‐automated cell‐counting tool. A total of 967 astrocytes were analyzed across six slices from three mice, and three distinct labeling profiles were identified: 63.81% of astrocytes were positive for S100b only, 13.34% showed co‐localization of anti‐S100b and anti‐GFP (GCaMP6s), and 22.8% were GFP‐positive but S100b‐negative (Figure 1C). Given that not all astrocytes express decent amounts of S100b to be detected by immunohistochemistry and that all GFP‐positive cells exhibited characteristic astrocytic morphology, we estimate the recombination efficiency of the GLAST‐CreERT2 × GCaMP6s^fl/fl^ line to be approximately 36.14% (sum of S100b^+^/GFP^+^ and GFP^+^ only cells).

**FIGURE 2:**
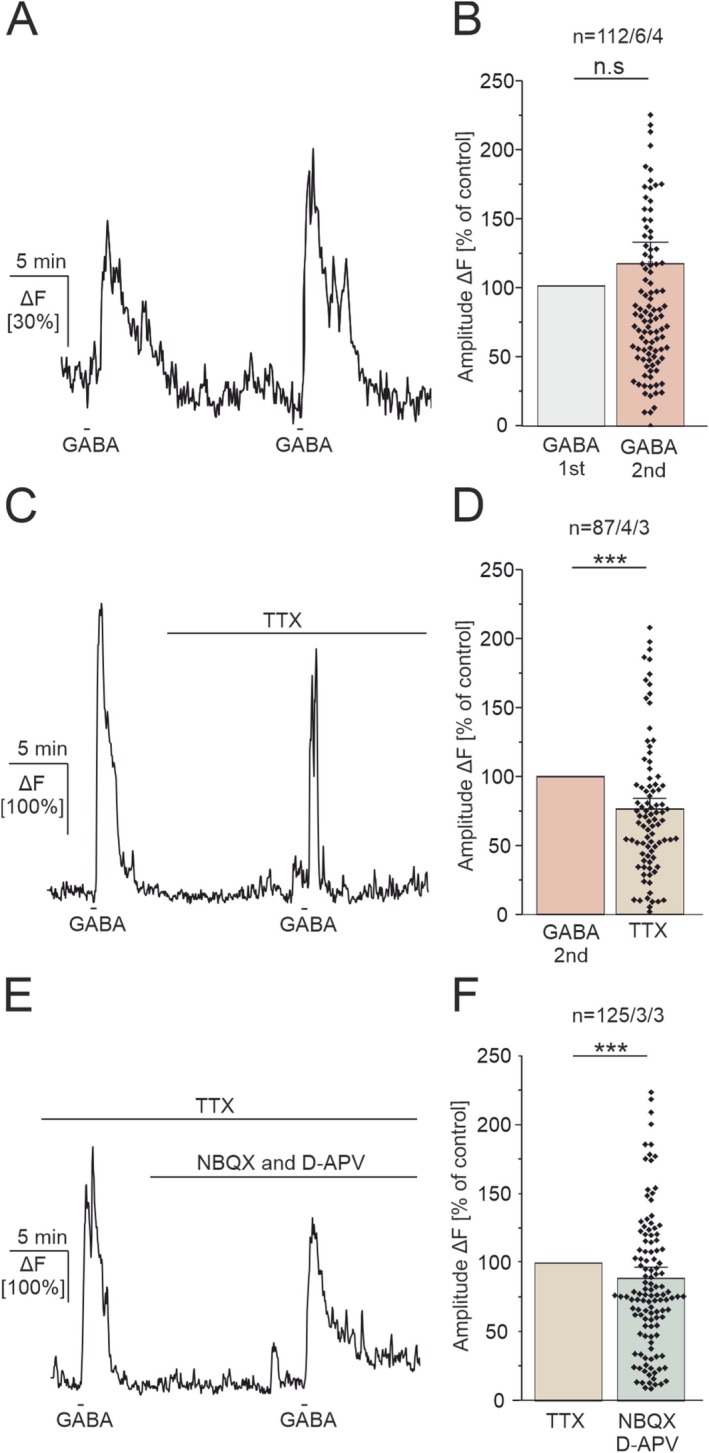
GABA‐induced Ca^2+^ transients in astrocytes are reduced in the presence of neuronal inhibition. (A) GABA‐induced Ca^2+^ transients are slightly increased upon recurring GABA application (“run‐up”). (B) Average amplitudes of GABA‐induced Ca^2+^ responses for the 1st GABA application (GABA 1st, gray bar) compared with the 2nd GABA application (GABA 2nd, red bar). (C) GABA‐induced Ca^2+^ transients are reduced in the presence of TTX (inhibitor of voltage‐gated sodium channels, 0.5 μM). (D) Average amplitudes of GABA‐induced Ca^2+^ responses in control conditions (the 2nd GABA application of the control experiment in Parts (A,B) served as a comparison for (C,D) and in the presence of TTX. (E) GABA‐induced Ca^2+^ transients are further reduced in the presence of glutamatergic inhibitors (D‐APV, 100 μM and NBQX, 10 μM). (F) Average amplitudes of GABA‐induced Ca^2+^ responses in control conditions; the GABA application in TTX (C,D) served as a comparison for (E,F) and in the presence of D‐APV and NBQX. Statistical significance was determined by the Wilcoxon test for paired data sets (B). For unpaired data sets, the Mann–Whitney *U* test was used (D, F). The error probability *p* was ***p < 0.001.

To investigate GABA‐mediated Ca^2+^ responses in astrocytes we next performed confocal Ca^2+^ imaging experiments in acute PFC slices. Bath application of GABA induced Ca^2+^ signaling in astrocytes in layer (L) 1–3 of the PFC in a dose‐dependent manner (Figure 1D–G). As the cerebral cortex is highly innervated by norepinephrine‐releasing fibers projecting from the locus coeruleus, the majority of cortical astrocytes respond to norepinephrine (NE) with Ca^2+^ transients (Ding et al. 2013). Therefore, we used bath application of NE (NE, 10 μM, 30 s) triggering α1receptor‐mediated Ca^2+^ signaling in astrocytes to estimate the number of astrocytes responding to GABA compared with NE in the field of view (Figure S1). The number of astrocytes that responded to the application of NE was defined as 100% responding astrocytes (Fischer et al. 2021). Bath application of GABA (50 μM, 30 s) resulted in Ca^2+^ transients that amounted to 8.4% ± 1.43% ΔF in 16.13% of the astrocytes (Figure 1D–G). GABA (200 μM) induced Ca^2+^ transients that amounted to 53.02% ± 4.32% ΔF in an increasing number of 36.93% of the astrocytes (*p* = 0.0001). The application of 500‐μM GABA induced Ca^2+^ transients with an amplitude of 91.93% ± 6.15% ΔF in 39.52% of the astrocytes, showing a significant increase in amplitude compared with 200 μM GABA (*p* = 0.0001). The highest concentration of 1‐mM GABA induced Ca^2+^ transients with an amplitude of 152.01% ± 13.85% ΔF in 55.81% of the astrocytes, showing a significant increase compared with 500‐μM GABA (*p* = 0.0078). The results indicate that about half of the astrocyte population in L1–3 of the PFC that express GCaMP6s (and responded to NE) also respond to the application of GABA with Ca^2+^ transients. For half‐maximal activation of GABA receptor‐mediated Ca^2+^ transients in our experiments a concentration of 500 μM GABA was sufficient to induce robust Ca^2+^ signals in a reasonable number of astrocytes of the PFC. To evaluate the possibility of GABA‐mediated receptor desensitization upon multiple GABA applications, we next performed two GABA applications with a 10‐min interval (rundown experiment). By comparing the second to the first GABA application, GABA‐induced Ca^2+^ responses in astrocytes showed an increase in amplitude to 115.3% ± 10.11% of the control, which was not significant (*n* = 112/6/4; *p* = 0.16 (Figure 2A,B). In the following, the rundown experiment (GABA 2nd) served as a control group. To elucidate any indirect effects on GABA‐induced Ca^2+^ transients in astrocytes we performed experiments in the presence of TTX (0.5 μM), suppressing action potential firing by neurons and thereby reducing neuronal transmitter release. GABA‐induced Ca^2+^ transients were significantly reduced to 82.02% ± 5.99% of the control (2nd GABA) (*n* = 87/4/3; *p* = 3.21 × 10^−7^) in the presence of TTX, suggesting neuronal impact on GABA‐induced Ca^2+^ transients in astrocytes (Figure 2C,D). Additional inhibition of glutamatergic AMPA (NBQX, 10 μM) and NMDA receptors (D‐APV, 100 μM) further reduced GABA‐induced Ca^2+^ transients significantly (Figure 2E,F). GABA‐induced Ca^2+^ transients in astrocytes in the presence of glutamatergic inhibition amounted to 96.64% ± 7.71% of the control, showing a significant reduction in amplitude compared with GABA‐induced Ca^2+^ transients in the presence of TTX (*n* = 125/3/3; *p* = 1.12 × 10^−4^). In general, multiple GABA applications lead to an increase in amplitude of GABA‐mediated Ca^2+^ transients, which are reduced in the presence of the voltage‐gated sodium channel inhibitor, TTX, and glutamatergic inhibitors, suggesting indirect neuronal impact. To avoid indirect effects on GABA‐induced Ca^2+^ signaling, the following experiments were performed in a combination of all three antagonists.

### 3.2 GABA_B_ Receptors Mediate Ca^2+^ Transients in Astrocytes

**FIGURE 3:**
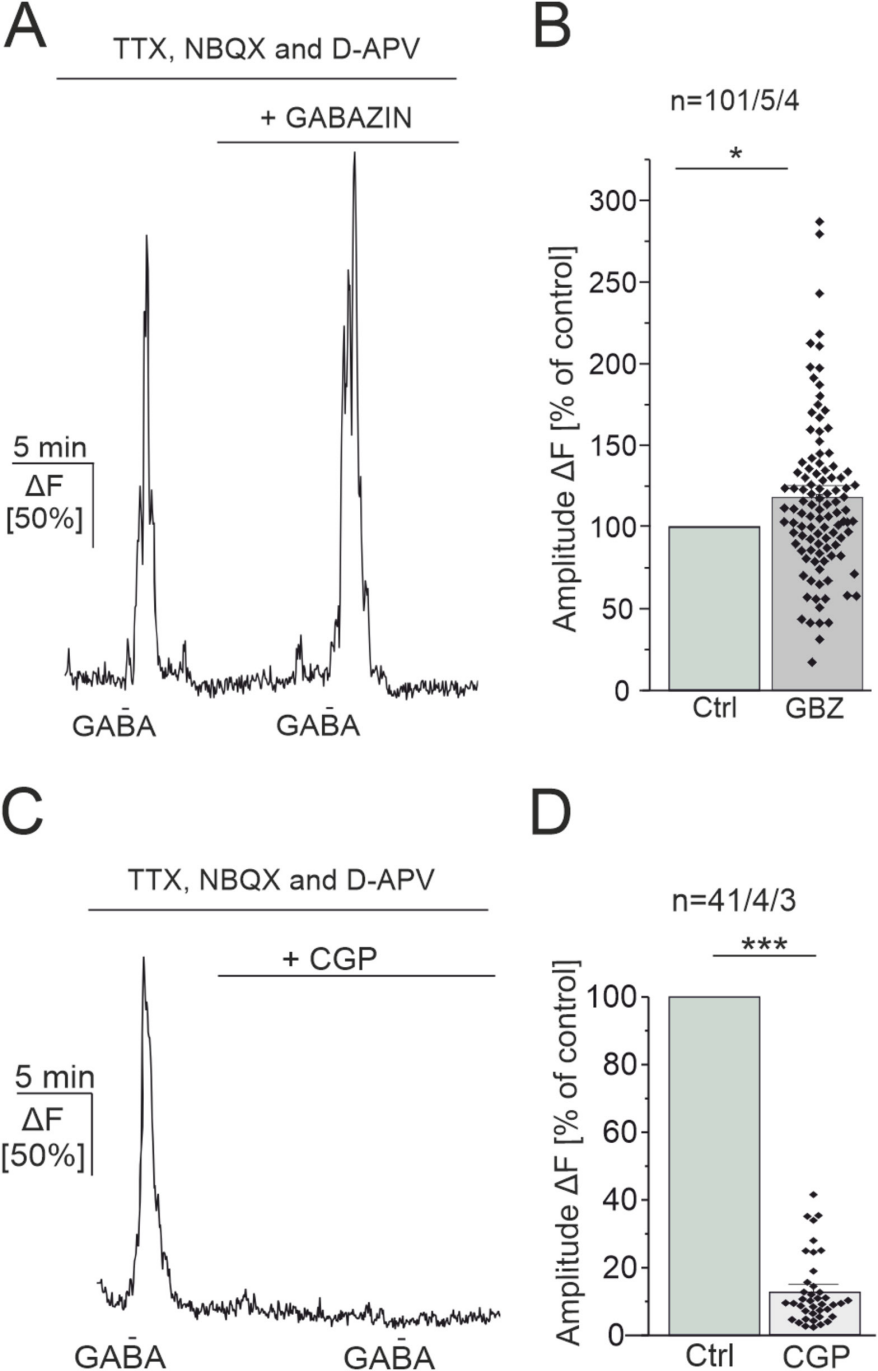
GABA‐induced Ca^2+^ transients in astrocytes are mediated by GABA_B_ receptors. (A) GABA‐induced Ca^2+^ transients are slightly enhanced in the presence of the GABA_A_ receptor antagonist, gabazine (5 μM). Note that these experiments were performed in the presence of TTX, D‐APV, and NBQX to isolate direct GABAergic Ca^2+^ responses in astrocytes with minimal neuronal influence. (B) Average amplitudes of GABA‐induced Ca^2+^ transients normalized to the control application. (C) GABA‐induced Ca^2+^ transients are strongly reduced in the presence of the GABA_B_ antagonist, CGP 55845 (10 μM). Average amplitudes of GABA‐induced Ca^2+^ transients normalized to the control application, showing a significant difference in the presence of the GABA_B_ receptor inhibitor, CGP 55845. Statistical significance was determined by the Wilcoxon test for paired data sets. The error probability *p* was **p < 0.01; ***p < 0.001.

Various signaling cascades have been reported leading to GABA‐mediated Ca^2+^ signals in astrocytes, greatly varying across experimental conditions, brain regions and developmental stages (Ishibashi et al. 2019). Both activation of the ionotropic GABA_A_ receptor and the metabotropic GABA_B_ receptor led to Ca^2+^ elevations in culture and in astrocytes of the somatosensory cortex and hippocampus (Mariotti et al. 2016; Meier et al. 2008; Nilsson et al. 1993). Moreover, GABAergic Ca^2+^ transients in developing olfactory bulb astrocytes depend on the reduced Na^+^/Ca^2+^ exchanger activity induced by loading astrocytes with Na^+^ upon GABA uptake (Doengi et al. 2009). To identify the receptors involved in GABA‐mediated Ca^2+^ signaling in PFC astrocytes, we tested the effect of the GABA_A_ receptor antagonist, gabazine (5 μM) and the GABA_B_ receptor antagonist, CGP 55845 (10 μM) on GABA‐induced Ca^2+^ responses in astrocytes. In the presence of gabazine, the amplitude of GABA‐induced Ca^2+^ responses significantly increased to 118.09% ± 4.84% of the control (*n* = 101/5/4; *p* = 0.03) (Figure 3A,B). The GABA_B_ receptor antagonist, CGP 55845 on the other hand nearly completely abolished GABA‐induced Ca^2+^ transients in astrocytes. The average amplitude of GABA‐induced Ca^2+^ transients in the presence of CGP 55845 amounted to 17.90% ± 4.09% of the control (*n* = 41/4/3; *p* = 1.11 × 10^−14^). The results show that GABA‐induced Ca^2+^ transients in astrocytes of the PFC are mediated by GABA_B_ receptors (Figure 3C,D).

### 3.3 GABA_B_‐Receptor Mediated Ca^2+^ Transients in Astrocytes Depend on the PLC/IP_3_‐Mediated Signaling Pathway

**FIGURE 4:**
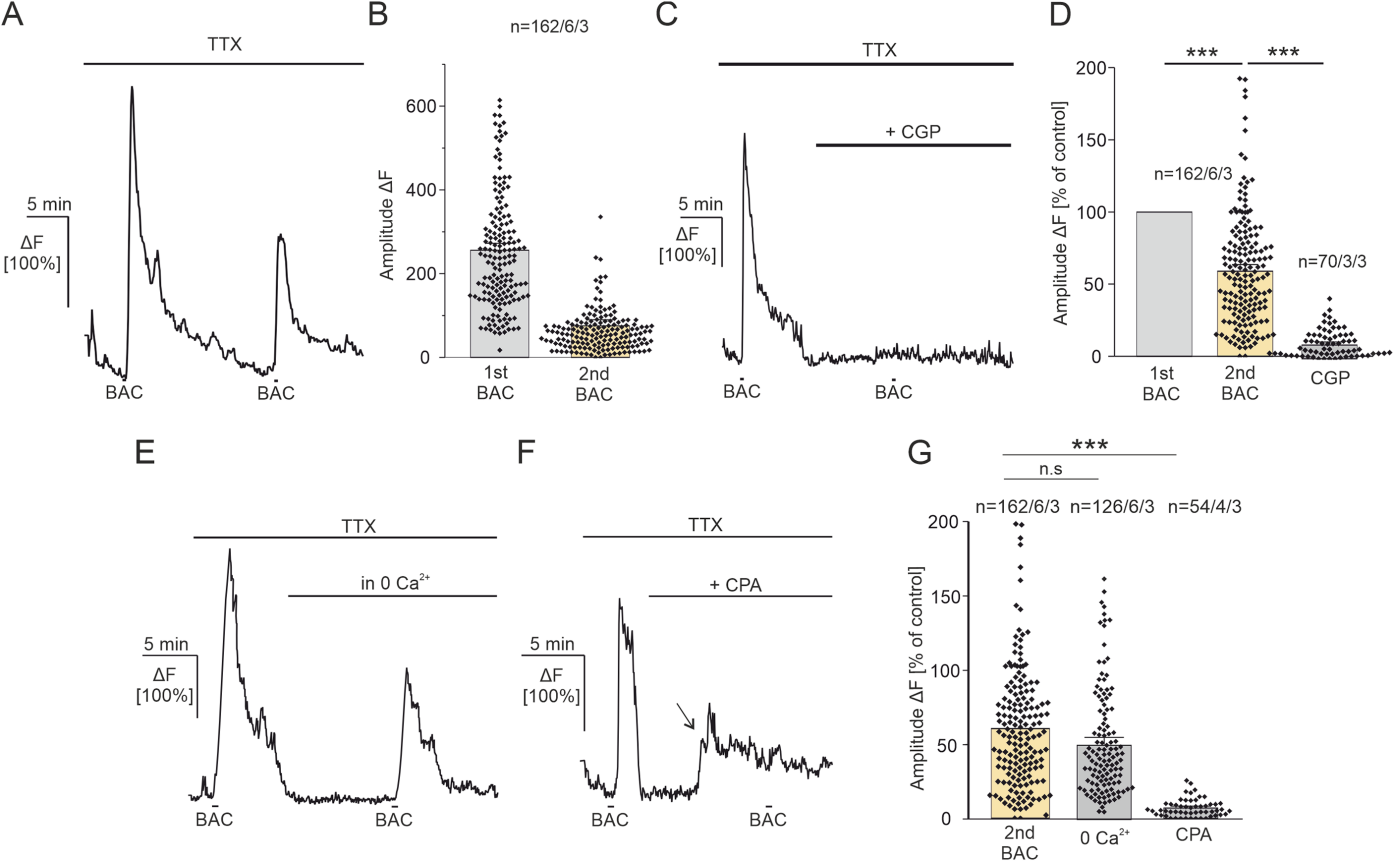
Baclofen‐induced Ca^2+^ transients in astrocytes depend on intracellular Ca^2+^ stores. (A) Baclofen‐induced Ca^2+^ transients are decreased upon recurring baclofen applications (“run‐down”). (B) Average amplitudes of baclofen‐induced Ca^2+^ responses for the 1st baclofen application (1st BAC, gray bar) compared with the 2nd BAC application (2nd BAC, yellow bar). The 2nd BAC application served as a control for the following experiments. (C) Baclofen‐induced Ca^2+^ transients are inhibited in the presence of the GABA_B_ receptor antagonist, CGP 55845 (10 μM). (D) Average amplitudes of baclofen‐induced Ca^2+^ responses in astrocytes normalized to the 1st BAC application. (E) Baclofen‐induced Ca^2+^ transients are not affected by the removal of extracellular Ca^2+^. (F) Baclofen‐induced Ca^2+^ responses are abolished in the presence of the SERCA pump inhibitor, CPA (20 μM). The arrow indicates Ca^2+^ store depletion upon SERCA pump inhibition. (G) Average amplitudes of baclofen‐induced Ca^2+^ transients in different conditions compared with the 2nd BAC application of the control (rundown) experiment. Statistical significance was determined by the Mann–Whitney *U* test for unpaired data sets (G). For paired data sets the Wilcoxon test was used (1st BAC vs. 2nd BAC). The error probability *p* was ***p < 0.001; n.s. = not significant.

Usually GABA_B_ receptors are G_i/o_‐GPCR‐coupled, inducing slow inhibitory signaling pathways in neurons. Presynaptically, GABA_B_ receptor‐mediated inhibition of voltage‐gated Ca^2+^ channels reduces neurotransmitter release, whereas postsynaptically GABA_B_ receptor‐activation recruits inwardly rectifying potassium channels resulting in a slow hyperpolarization of the cell. In astrocytes, GABA_B_ receptor‐activation initiates Ca^2+^ responses, suggesting the involvement of the G_q_‐GPCR‐signaling cascade (Mederos et al. 2021). However, in astrocytes of the somatosensory cortex of juvenile mice (p15‐p20), GABA_B_ receptor‐mediated Ca^2+^ responses involve both the G_q_‐ and the G_i_‐signaling cascades (Mariotti et al. 2016). We aimed to investigate the intracellular signaling pathway that leads to GABA_B_ receptor‐mediated Ca^2+^ transients in astrocytes of the young adult PFC. Therefore, we applied the specific GABA_B_ receptor agonist, baclofen to induce robust GABA_B_ receptor‐specific Ca^2+^ transients. Bath application of baclofen (200 μM) evoked Ca^2+^ transients in astrocytes with an average amplitude of 220.72% ± 11.77% ΔF. We performed multiple applications of baclofen with a 10‐min interval in‐between to evaluate a possible decrease in baclofen‐induced Ca^2+^ transients by receptor or signaling cascade desensitization (rundown). The second application of baclofen induced Ca^2+^ transients amounted to 59.70% ± 3.02% of the first baclofen application (control) and showed a significant reduction (*n* = 162/6/3; *p* = 0.001, Figure 4A,B). Therefore, in the following, baclofen‐induced Ca^2+^ transients in astrocytes in presence of different antagonists are compared with the corresponding baclofen application in the rundown experiment (2nd BAC). To control the agonist specificity of baclofen, we applied baclofen in the presence of the GABA_B_ receptor antagonist, CGP 55848. In the presence of CGP 55848, baclofen‐induced Ca^2+^ transients in astrocytes were highly and significantly reduced and amounted to 10.3% ± 1.11% of the control, showing specific activation of GABA_B_ receptors in astrocytes (*n* = 70/3/3; *p* = 2.41 × 10^−34^, Figure 4C,D).

**FIGURE 5:**
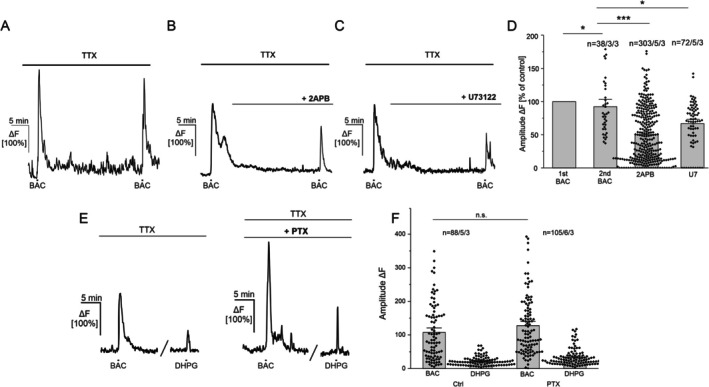
Baclofen‐induced Ca^2+^ transients depend on the PLC/IP_3_ signaling pathway. For antagonizing different compartments of the intracellular signaling cascade, antagonists need to enter the cells, which may take longer in our experimental settings. Therefore we performed corresponding rundown/control experiments, with a 30‐min interval between the two baclofen applications. (A) Ca^2+^ transients upon repetitive baclofen applications show a small reduction. (B) Baclofen‐induced Ca^2+^ transients are decreased in the presence of the IP3‐receptor antagonist, 2APB (100 μM). (C) Baclofen‐induced Ca^2+^ transients are decreased in the presence of the PLC‐antagonist, U73122 (50 μM). (D) Average amplitudes of baclofen‐induced Ca^2+^ responses in astrocytes in the presence of the different antagonists normalized to the 1st BAC application. (E) Baclofen‐ and DHPG‐induced Ca^2+^ transients in control conditions (preincubation in ACSF; first panel) and with a 4‐h preincubation with the Gi‐protein inhibitor, PTX (7.5 μg/mL). Average amplitudes of baclofen‐ and DHPG‐induced Ca^2+^ transients in control conditions and in PTX. Statistical significance was determined by the Mann–Whitney *U* test for unpaired data sets. The error probability *p* was *p < 0.05; ***p < 0.001; n.s. = not significant.

To investigate the signaling pathway responsible for GABA_B_ receptor‐mediated Ca^2+^ transients in astrocytes, we first removed extracellular Ca^2+^. In Ca^2+^‐free ACSF (0 Ca^2+^), baclofen induced Ca^2+^ transients that amounted to 49.90% ± 2.49% of the control and were not significantly different compared with the rundown (*n* = 126/6/3; *p* = 0.09), suggesting a minor impact of extracellular Ca^2+^ (Figure 4E,G). Intracellular Ca^2+^ store depletion by the inhibition of the SERCA pumps by cyclopiazonic acid (CPA, 20 μM), on the other hand, almost completely abolished baclofen‐induced Ca^2+^ transients in astrocytes. Baclofen‐induced Ca^2+^ transients in the presence of CPA showed reduced amplitudes that amounted to 6.98% ± 0.81% of its control, showing a significant contribution of intracellular Ca^2+^ stores on GABA_B_ receptor‐mediated Ca^2+^ signaling (*n* = 54/4/3; *p* = 1.21 × 10^−32^; Figure 4F,G). It should be noted that Ca^2+^ store depletion by SERCA‐pump inhibition using CPA itself induces a rise in intracellular Ca^2+^ levels (Figure 4F, arrow). We further elucidated the involvement of the phospholipase C (PLC) and IP_3_‐receptors in the signaling cascade by using specific antagonists. In the presence of the IP_3_‐receptor antagonist, 2APB (100 μM) baclofen‐induced Ca^2+^ transients amounted to 48.92% ± 2.29% of the control and were significantly reduced compared with the rundown (*n* = 303/5/3; *p* = 9.11 × 10^−5^; Figure 5B,D). To compare these experiments, we performed an additional rundown experiment, matching the timeline of the performed experiment with a 30‐min application for antagonists with intracellular targets. In the corresponding rundown experiment, baclofen‐induced Ca^2+^ transients amounted to 92.23% ± 7.19% of its control (*n* = 38/3/3; *p* = 0.02) (Figure 5A,D). Moreover, the application of the PLC‐inhibitor U73122 (50 μM) also reduced baclofen‐induced Ca^2+^ transients in astrocytes to 77.64% ± 5.04% of its control (*n* = 72/5/3; *p* = 0.03) (Figure 5C,D). The results show that GABA_B_ receptor‐mediated Ca^2+^ transients in astrocytes greatly depend on intracellular Ca^2+^ stores triggering the PLC/IP_3_‐signaling cascade. Next, we tested the involvement of the G_i/o_ signaling cascade on GABA_B_‐receptor mediated Ca^2+^ signaling in astrocytes. Therefore, we studied baclofen‐induced Ca^2+^ responses in the presence of the G_i/o_ protein inhibitor, pertussis toxin (PTX, 7.5 μg/mL). Slices were pre‐incubated with PTX in ACSF for 4 h. Besides baclofen, we applied the mGluR‐Agonist, DHPG, to induce Ca^2+^ responses insensitive to PTX (Mariotti et al. 2016). We compared these with baclofen‐ and DHPG‐induced Ca^2+^ transients in control conditions (4‐h preincubation in ACSF without PTX). In the control group, baclofen induced Ca^2+^ transients that amounted to 107.44% ± 8.9% ΔF (*n* = 88/5/3). DHPG induced Ca^2+^ transients that amounted to 22.56% ± 1.5% ΔF. In the presence of PTX, both baclofen‐ and DHPG‐induced Ca^2+^ transients in astrocytes increased to 129.1% ± 8.5% ΔF for baclofen and to 30.32% ± 2.42% ΔF for DHPG (*n* = 105/6/3, *p* = 0.06; Figure 5E,F). Baclofen‐induced Ca^2+^ transients showed no significant difference in control conditions and in the presence of the G_i/o_ protein inhibitor, PTX. Our results suggest no direct impact of the G_i/o_ protein on GABA_B_receptor‐mediated Ca^2+^ signaling in young adult PFC astrocytes.

## 4 Discussion

In the present study, we investigated GABA‐mediated Ca^2+^ signaling in astrocytes of the PFC of young adult mice. Moreover, we deciphered the intracellular signaling cascade leading to the GABA‐induced rise in the intracellular Ca^2+^ concentration in astrocytes. Our results demonstrate that GABA_B_ receptor‐mediated Ca^2+^ transients in astrocytes of the young adult PFC depend on the G_q_‐signaling cascade and the Ca^2+^ release from intracellular Ca^2+^ store upon PLC and IP_3_ receptor activation, rather than on the G_i_‐signaling cascade.

### 4.1 GABA_B_ Receptor‐Mediated Ca^2+^ Signaling in Astrocytes Depend on the G_q_‐Protein Coupled Signaling Pathway

In this study, we employed a commonly used Cre/LoxP‐based mouse line that expresses the genetically encoded calcium indicator GCaMP6s under control of the astrocyte‐specific GLAST‐promoter to investigate GABA‐driven calcium dynamics in astrocytes. Immunohistochemical analysis revealed high specificity of GCaMP6s expression in astrocytes, as indicated by co‐localization with the astrocyte marker S100b and characteristic astrocytic morphology. The recombination efficiency was estimated at approximately 36.14%, supporting the suitability of this animal model for the present study. In astrocytes the most common Ca^2+^ signaling mechanism is the G_q_‐GPCR‐coupled pathway, resulting in the IP_3_‐dependant Ca^2+^ release from internal stores, just like the endoplasmic reticulum. As a consequence of astrocytic Ca^2+^ elevations, gliotransmitters are released, acting on neighboring neurons influencing neuronal network activity and animal behavior (Araque et al. 1998; Bezzi et al. 1998; Perea and Araque 2007; Mederos et al. 2021). Performing confocal Ca^2+^ imaging in acute PFC slices, we show that about 56% of the astrocytes in the adult PFC, that underwent Cre‐dependent GCaMP expression, respond to bath application of GABA, suggesting that only a subpopulation of astrocytes is able to respond to GABA with Ca^2+^ elevations. This is in line with the general view that astrocytes are a highly heterogenous cell population, greatly differing in morphology, receptor expression profile, and Ca^2+^ signaling dynamics even within brain regions (Pestana et al. 2020). Although, GABA‐induced Ca^2+^ responses show a dose–response relationship, the applied concentrations of GABA may not be constant in the perfusion bath, as extracellular GABA is a dynamic signaling molecule, which is also rapidly taken up by GABA transport mechanisms of neurons and astrocytes, without being able to develop its full effect on the receptor. GABA‐induced Ca^2+^ signaling in astrocytes show a slight but significant reduction upon inhibition of neuronal action potential firing (using TTX), which seems not intuitive in the first place, as GABA induces neuronal hyperpolarization rather than depolarization and neurotransmitter release. However, GABA‐mediated Ca^2+^ release of astrocytes may lead to the release of gliotransmitters, such as glutamate or ATP (Mederos et al. 2021), inducing secondary neuronal depolarization, leading to signal amplification in astrocytes, which would be abolished in the presence of TTX or glutamatergic inhibitors. Classically, neither the ionotropic GABA_A_ receptor, nor the metabotropic GABA_B_ receptor are G_q_‐coupled receptors. Whereas GABA_A_ receptors act as chloride channels, inducing a chloride efflux and depolarization in astrocytes (Untiet et al. 2024), the GABA_B_ receptor is G_i/o_‐GPCR‐coupled reducing the intracellular cAMP concentration by adenylyl cyclase activity reduction. However, astrocytes have been shown to express both GABAreceptor subtypes, both being linked to intracellular Ca^2+^ signaling (Kang et al. 1998; Mederos et al. 2021; Meier et al. 2008; Serrano et al. 2006). Additionally, GABA‐induced Ca^2+^ signaling upon GABA transporter activity has also been reported in astrocytes of the developing olfactory bulb (Doengi et al. 2009). Thus, there seem to be various mechanisms leading to functional GABAergic Ca^2+^ signaling in astrocytes depending on brain region and from development to adulthood. In this study we demonstrate that GABA‐induced Ca^2+^ transients in astrocytes of the young adult PFC are not decreased in the presence of the GABA_A_receptor antagonist gabazine but significantly reduced by the GABA_B_receptor antagonist, CGP 55848. Consequently, GABA_B_ receptors are crucial for GABA‐mediated Ca^2+^ signaling in the PFC. This is in line with results obtained from the developing somatosensory cortex and hippocampus (Mariotti et al. 2016; Meier et al. 2008). In accordance to that, astrocyte‐specific GABA_B_ receptor *knock‐out* mice show no Ca^2+^ elevations upon baclofen application (Cheng et al. 2023). Additionally, we show that GABA_B_ receptor‐mediated Ca^2+^ transients are diminished after intracellular store depletion by inhibition of the SERCA pumps with CPA, indicating the contribution of intracellular Ca^2+^ stores. Hence, the involvement of the PLC/IP_3_‐signaling cascade upstream to the Ca^2+^ release from intracellular Ca^2+^ stores is likely. Moreover, our results show a reduction of GABA_B_ receptor‐mediated Ca^2+^ signaling after pharmacological inhibition of both the IP_3_‐receptor (2APB) and the PLC (U73122), underlining the necessity of the G_q_‐GPCR‐signaling pathway for GABAergic Ca^2+^ signaling in astrocytes of the PFC. It should be noted, that both, the PLC and IP_3_ receptor antagonist need to reach intracellular targets, which may account for the big variability of the baclofen‐induced Ca^2+^ signals in the presence of these antagonist. Additionally, one limitation of the conducted experiments is that all antagonists used, do not target and effect astrocytes specifically but influence the entire cellular network. However, in line with our results, GABA‐mediated Ca^2+^ oscillations in the somatosensory cortex are greatly reduced in IP_3_R2 *knock‐out* mice (Mariotti et al. 2016), underlining the impact of the IP_3_R mediated signaling cascade for GABA_B_ receptor‐mediated Ca^2+^ responses in astrocytes. On the other hand, in the same study the G_i/o_ protein antagonist PTX reduced GABA_B_‐receptor mediated Ca^2+^ responses in astrocytes of the developing somatosensory cortex, arguing that both, the G_q_‐ and G_i/o_‐pathway might be linked for functional GABAergic Ca^2+^ signaling in astrocytes of the cortex. Favoring this hypothesis, it has been shown that chemogenetic G_i/o_‐GPCR activation leads to Ca^2+^‐signaling in astrocytes of the hippocampus as well (Durkee et al. 2019). Contrary, our results show no effect of the G_i/o_ inhibitor, PTX on GABA_B_receptor‐mediated Ca^2+^ signaling in astrocytes of the young adult PFC, suggesting the sole involvement of the G_q_‐signaling cascade in adolescence and presumably in adulthood. Just recently, it has been reported that GABA_B_receptor activation in cortical astrocytes induces robust Ca^2+^ transients, but a relative lack of cAMP dynamics, indicating that at least in adult mice the classical G_i/o_ signaling cascade leading to cAMP reduction upon GABA_B_ receptor activation in astrocytes, is less prominent (Cahill et al. 2024). GABA_B_receptors have been shown to be critical regulators of astrocytogenesis, as the astrocyte‐specific depletion of GABA_B_ receptors in the developing cortex results in abnormal astrocyte morphology (Cheng et al. 2023). In light of this, it is plausible that the signaling cascade underlying GABA_B_receptor‐mediated Ca^2+^ signaling in cortical astrocytes might undergo developmental changes from early development to adulthood. Specifically, the signaling cascade may involve the G_q_ and G_i/o_ pathways during development, shifting to the G_q_‐pathway only in adulthood. However, this would need further investigations such as a direct comparison of the signaling cascades at different developmental stages.

## Author Contributions

**Jennifer Bostel:** formal analysis, investigation, writing – review and editing. **Alina J. Kürten:** formal analysis, investigation, writing – review and editing. **Antonia Beiersdorfer:** conceptualization, data curation, investigation, project administration, supervision, writing – original draft, writing – review and editing.

## Conflicts of Interest

The authors declare no conflicts of interest.

## Peer Review

The peer review history for this article is available at https://www.webofscience.com/api/gateway/wos/peer‐review/10.1111/ejn.70187.

## Supporting information


**Figure S1:** GABA and Norepinephrine (NE) induce Ca^2+^ signaling in PFC astrocytes. (A) Confocal image of GCaMP6s expression in astrocytes of the PFC. Scale bar: 200 μm. (B) Astrocytes respond to GABA application (500 μM) with Ca^2+^ transients. (C) Virtually most astrocytes in the field of view respond to NE application (10 μM) with Ca^2+^ transients. The arrowhead indicates the ROI depicted in D. The arrow indicates the ROI depicted in E. (D) An example trace of an astrocyte that responds to GABA and NE. (E) An example trace of an astrocyte only responding to NE and not to GABA.

## Data Availability

All data of this study will be made available by the authors upon request.
