# Supplementary material for: GABAB Receptors Mediate Intracellular Calcium Release in Astrocytes of the Prefrontal Cortex
Source: Eur J Neurosci. 2025 Jul 14;62(1):e70187. doi: 10.1111/ejn.70187 (PMC12257579; doi:10.1111/ejn.70187)
Supplement: Supplementary file 1 — Figure S1: GABA and Norepinephrine (NE) induce Ca2+ signaling in PFC astrocytes. (A) Confocal image of GCaMP6s expression in astrocytes of the PFC. Scale bar: 200 μm. (B) Astrocytes respond to GABA application (500 μM) with Ca2+ transients. (C) Virtually most astrocytes in the field of view respond to NE application (10 μM) with Ca2+ transients. The arrowhead indicates the ROI depicted in D. The arrow indicates the ROI depicted in E. (D) An example trace of an astrocyte that responds to GABA and NE. (E) An example trace of an astrocyte only responding to NE and not to GABA. [file EJN-62-0-s001.docx]

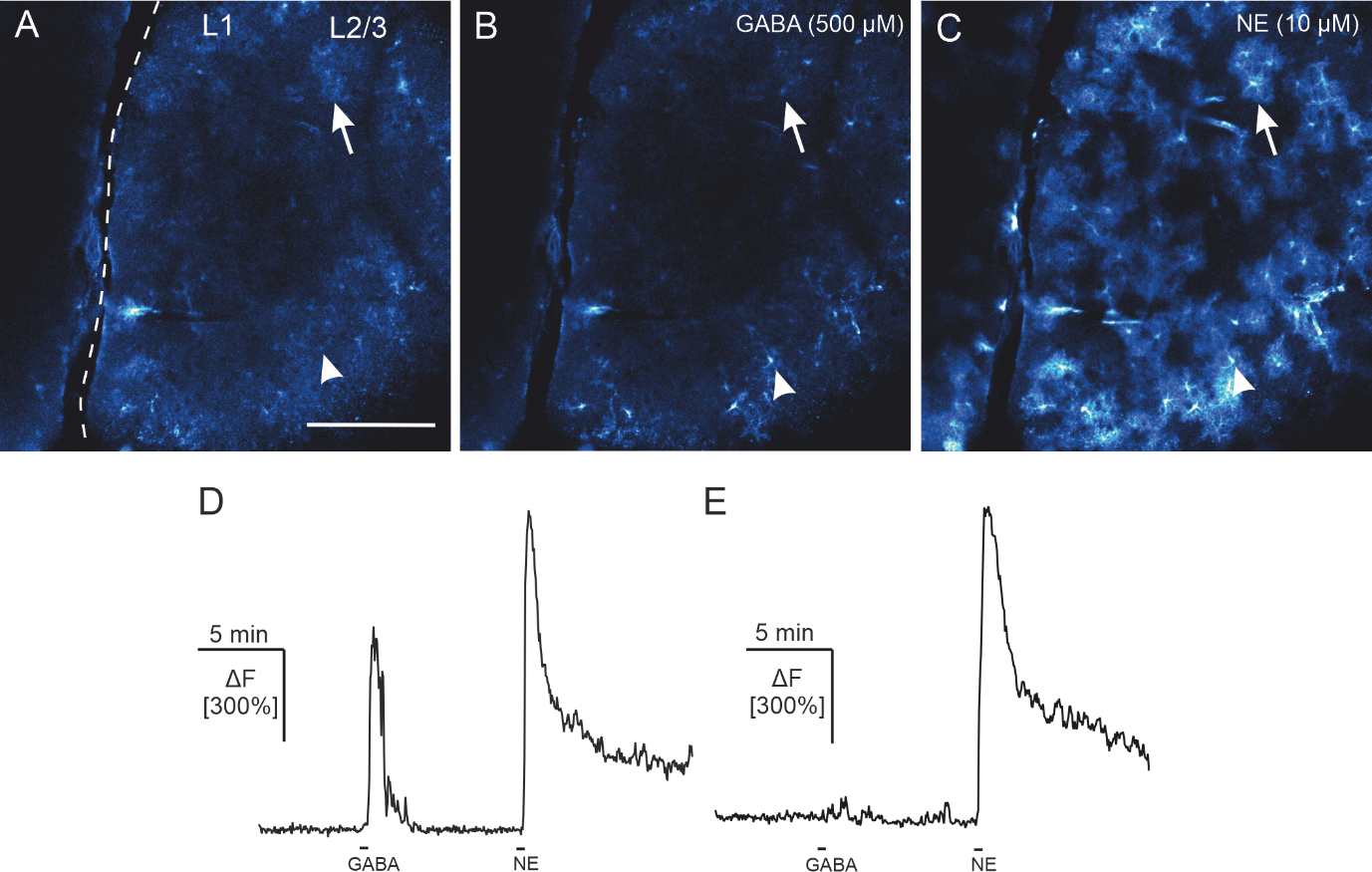


**Suppl. Figure 1:** GABA and Norepinephrine (NE) induce Ca^2+^ signaling in PFC astrocytes. (A) Confocal image of GCaMP6s expression in astrocytes of the PFC. Scale bar: 200 µm. (B) Astrocytes respond to GABA application (500 µM) with Ca^2+^ transients. (C) Virtually most astrocytes in the field of view respond to NE application (10 µM) with Ca^2+^ transients. The arrowhead indicates the ROI depicted in D. The arrow indicates the ROI depicted in E. (D) An example trace of an astrocyte that responds to GABA and NE. (E) An example trace of an astrocyte only responding to NE and not to GABA.
